# Prognostic factors in bushfire‐affected koalas–Kangaroo Island bushfire response 2020

**DOI:** 10.1111/avj.13434

**Published:** 2025-03-12

**Authors:** O Funnell, J McLelland, A Kokosinska, T Stephenson, E Dunstan, B Turner, N Speight

**Affiliations:** ^1^ Zoos SA Adelaide Australia; ^2^ School of Animal and Veterinary Sciences, Faculty of Sciences, Engineering and Technology University of Adelaide Roseworthy Australia

**Keywords:** burns, bushfire, koala, prognostic factor, triage, wildlife

## Abstract

This study presents a retrospective analysis of clinical records data from koalas presenting for treatment following the large‐scale bushfire event on Kangaroo Island 2019–2020. The aim of the study was to identify prognostic factors for koalas affected by bushfire. Koalas (n = 199) were grouped based on their burn status in combination with their burn bandaging requirement at triage; those with no burns, with burns that did not require bandaging and with burns that did require bandaging. Overall, 59.8% of koalas had positive outcomes, but this reduced to only 13% of koalas in the most severely affected group (burns that were bandaged). Negative outcomes were found to be associated with several factors, including the timing of presentation, with the worst affected animals presenting in the first 2 weeks of the operation. Also, an increasing number of bandage changes during hospitalisation led to increasingly negative outcomes, with no positive outcomes beyond three bandage changes. In addition, clear associations were found with patterns of burns on feet, with only 4% positive outcomes for animals with all 4 feet burnt. All bandaged koalas with severe dehydration had negative outcomes and body weights decreased over time for hospitalised animals from the most severely affected bandaged burn group. Mild serum sodium concentration elevation at triage was the only clinically significant blood abnormality for koalas with bandaged burns. Overall, this study identified key prognostic factors, particularly bandaging requirements and the number of feet burnt, that will enable more effective decision‐making at triage and improve animal welfare outcomes.

**Abbreviations**
ALT: alanine transaminase
AST: aspartate transaminase
BCS: body condition score
CDTS: caeco‐colic dysbiosis/Typhlocolitis syndrome
CK: creatine kinase
DNA: deoxyribonucleic acid
EDTA: ethylenediaminetetraacetic acid
GGT: gamma‐glutamyltransferase
Hct: haematocrit
KIWP: Kangaroo Island Wildlife Park
KoRV: koala retrovirus
MCH: mean corpuscular haemoglobin
MCHC: mean corpuscular haemoglobin concentration
MCV: mean corpuscular volume
NSAID: non‐steroidal anti‐inflammatory drug
PhaHV: phascolarctid gammaherpesvirus
SAVEM: South Australian Veterinary Emergency Management
SR: soft release
WCC: white cell count

The koala (*Phascolarctos cinereus*) is frequently presented for treatment following bushfire events.^1^, ^2^ In regions where this species is abundant, koalas can represent a significant proportion of wildlife casualties presented for post‐bushfire treatment.^1^, ^3^

Kangaroo Island (KI) is the third largest island off the Australian coast, with an area of 440,500 ha, and is considered an important conservation area. The 2019/2020 bushfires on KI burnt an estimated 200,000 ha, including 96% of the Flinders Chase National Park and adjoining Ravine des Casoars, 98% of the Kelly Hill Conservation Park and most of the mature Tasmanian blue gum plantations.^4^, ^5^ This resulted in 85% of the known koala habitat being fire affected, with suggestions that the island's koala population of an estimated 50,000 was reduced to between 5 and 10,000 animals.^4^ A 2015 survey of KI estimated nearly half of the koalas (23,360 ± 3330) resided in the gum plantations.^6^ The KI population likely represented one of the largest and densest populations of koalas in Australia prior to the fires. Mass casualty events involving wildlife are not common; however, a large‐scale bushfire can affect large numbers of animals and quickly overwhelm local facilities. The scale of the KI event, the low human population and the high density of wildlife resulted in large numbers of casualties presenting to one location.

Triage (from the French word ‘Trier’ – to choose, pick out or sift) originated from sorting casualties in battlefield situations^7^ and is a dynamic process using scales and scores alongside physiological measurements and clinical examinations. Molony et al.^8^ suggest that there is no clear consensus on how to triage wildlife casualties, with decisions often being based on speculation regarding the chances of release. Factors influencing the decision to treat or euthanase, or the treatment given, may include clinician expertise, resources, philosophy (e.g. give every individual a chance), welfare considerations, captive options or release possibility. Additionally, the lack of guidance on what factors will influence outcome, as well as unclear definitions of what a successful outcome entails, will also lead to inconsistent decision‐making.

Following a systematic review of the available literature, Cope et al.^9^ found that evidence‐based, species‐specific and context‐specific protocols are necessary to maximise positive outcomes for wildlife entering rehabilitation. Development of protocols will also help responders to mass casualty events to make rescue and treatment decisions quickly and consistently, as well as direct resources in a time‐sensitive and efficient manner.^9^

For koalas, prognostic factors influencing outcome are currently not well understood, and guidelines for decision‐making not readily available during an event. Clark et al.^10^ define a prognostic factor as ‘a measurement that is associated with a clinical outcome in the absence of therapy or with the application of a standard therapy that all patients are likely to receive’.

In an initial study of koalas rescued from the Kangaroo Island fires, Dunstan et al.^2^ examined records at the point of triage, looking at demographics, examination data and outcomes, and concluded that burn severity was the most important prognostic indicator. Body condition score and hydration status of animals at presentation were also useful initial assessments. However, this previous study did not examine positive outcomes for koalas based on hospitalisation and administered treatments; hence, in the current study, we examine the prognostic factors for the koalas that entered care past the point of triage, with an aim of improving triage and treatment protocols for koalas, leading to better welfare outcomes.

## Materials and methods

A total of 666 animals comprising 10 different species (with an estimated 565 koalas) were received over a period of 11 weeks, with formal recording of triage and hospital information between 9 January and 15 March 2020. During and following the fire, the Kangaroo Island Wildlife Park (35.7866° S, 137.2292° E) and its existing facilities were rapidly repurposed to receive and treat fire‐affected wildlife, despite the threat of fire to the site itself. The operation then shifted to an inflatable triage tent on the same site, deployed by the South Australian Veterinary Emergency Management group. As the response became more coordinated and the threat of fire decreased, a large canvas Australian Army tent and a demountable building were sourced and set up for triage and treatment of animals. Over the course of the response, facilities to house and hospitalise animals were constructed and two fenced and wooded ‘soft release’ areas were used for rehabilitating koalas.

### Inclusion criteria

Triage and hospital records were examined for completeness of data following the same criteria as Dunstan et al.^2^ resulting in 304 records. These records were further examined for animals that progressed past the point of triage with well‐described injuries and known outcomes, resulting in 199 available records. Animals euthanased at triage or with incomplete records were excluded (105 records).

### Record standardisation

‘Open style’ paper records were used during the operation resulting in multiple terms and schemes being used to describe burns, hydration status, age and body condition by 22 veterinarians. To standardise and group these descriptions, the previously published scales (outlined below) have been used for consistency.^2^

### Body condition score, age and hydration

Body condition (BCS) of koalas was standardised as poor (<3) or adequate (≥3) on a scale of 1–5.^11^ The ages of animals were grouped based on tooth wear classes: independent juveniles (TWC I: 1–2 years), young adults (TWC II‐III: 3–4 years) and adults (TWC IV: 5 years and above).^12^ Hydration estimates were scored, 0 if there was no evidence of dehydration, 1 for mild, 2 for moderate and 3 for severe.

### Burn classification and bandaging

Descriptions of burns were rationalised into four classes and assigned a burn severity score. Unburnt animals received a score of 0, superficial burns a score of 1, partial thickness a score of 2 and full thickness a score of 3.^13^ Mean burn score was calculated as the average of the burn scores from five body regions (head, left and right forelimb, left and right hindlimb), while maximum burn score refers to the score of the most severely burnt region.

Koalas were grouped based on the presence or absence of burns in conjunction with whether bandaging was applied at triage, so as to easily differentiate three groups: Group 1 – no detectable burn injury at triage, Group 2 – bushfire exposure evident at triage (ranging from singed haircoat to minor lesions) but no triage bandaging required, Group 3 – bushfire exposure resulting in significant burn injuries found at triage that required bandaging.

### Other data

Other data available from hospital records included triage date, duration of hospitalisation, drugs used during treatment, fluid therapy, bandaging regimes, weight changes during hospitalisation, time in the soft release area and outcomes (positive = release; negative = died or euthanased). In some individuals, blood samples were taken at triage for biochemical and haematological analysis, and swabs for chlamydial testing.

For this study, hospitalisation refers to the period when animals were contained in fabricated enclosures and closely monitored. Soft release refers to the time spent in two large fenced wooded areas away from the hospital with less monitoring. The term release is used to describe release back into the wild.

### Laboratory methods

Blood was analysed by Gribbles Veterinary Pathology (Glenside, South Australia), from 67/199 koalas, sampled either at or within 24 h of triage. Biochemical examination of serum using a Siemens Advia 1800 Clinical chemistry system was performed for the following analytes: alkaline phosphatase, inorganic phosphate, calcium, magnesium, total bilirubin, alanine transaminase, aspartate transaminase, gamma‐glutamyl transferase, creatinine kinase, total protein, albumin, globulins, sodium, potassium and chloride, and cortisol was measured with Immulite 2000 XPi.

Haematological parameters were analysed using whole blood preserved using EDTA in an Abbott Cell Dyn 3700 multiparameter haematology analyser for the following analytes: red cell count, haemoglobin, Hct, MCV, MCH, MCHC, WCC, neutrophils, lymphocytes, monocytes, eosinophils, basophils, fibrinogen and platelets.

A limited number of necropsies were performed on‐site, with tissue samples processed routinely for histopathological examination at the Veterinary Diagnostic Laboratory, University of Adelaide, or at Zoos SA.

### Molecular analyses

Where available, frozen stored whole blood samples were tested for koala retrovirus (KoRV). Any frozen stored ocular and urogenital swabs taken at triage were tested for *Chlamydia pecorum* and Phascolarctid gammaherpesvirus 1 and 2.

DNA was extracted using the QIAamp DNeasy Minikit (Qiagen, Hilgen, Germany) as per the manufacturer's instructions. Extracted DNA was measured by the NanoDrop One spectrophotometer (Thermo Fisher Scientific Inc, United States) and stored at −20°C until testing. KoRV proviral load was measured in DNA extracted from whole blood, and *C. pecorum* and Phascolarctid gammaherpesvirus 1 and 2 were tested in DNA extracted from ocular and urogenital swabs.

*C. pecorum* was detected by qPCR using primers from Hulse et al. 2018,^14^ KoRV *pol* gene qPCR with primers described in Tarlinton et al. 2005^15^ and run in triplicate. KoRV analysis qPCR conditions are described in Stephenson et al.^16^ PhaHV 1 and 2 were detected in PCR as per Kasimov et al.^17^ Koala β‐actin was used as the reference gene for quality control of extractions and normalisation of KoRV proviral load using primers described in Shojima et al.^18^ Samples were removed from the study if no β‐actin was detected.

### Statistical data analysis

To compare the three groups for continuous variables such as blood analyte results and body condition score, ANOVA tests were used for parametrically distributed data with post hoc t‐tests if significant. For non‐parametrically distributed data, Kruskal–Wallis comparison was used with post hoc Mann–Whitney U tests. Body weight data were analysed as the difference in weight at subsequent assessments from that recorded at triage (%) for the three koala groups. To determine significant correlations over time for each subsequent recorded body weight and other clinical variables with koala outcomes, Pearson's correlations were undertaken. Chi‐squared tests for association were used for comparing outcomes for categorical variables. Statistical significance was defined as P‐values of less than 0.05.

## Results

### Classification of koalas into groups based on burns and bandaging requirements

Records for 199 koalas met the inclusion criteria, and these were classified into Group 1 (no detectable burn injury at triage) with 61 koalas (31%), Group 2 (bushfire exposure evident at triage but no triage bandaging required) 77 koalas and Group 3 (significant burn injuries at triage requiring bandaging at triage) 61 koalas. Of the total 136/199 (69%) koalas that had burns, the mean maximum burn severity (out of 3) for Group 2 koalas for which burn severity was recorded (n = 46) was 0.9 ± 0.36 (SD) and the mean average burn severity was 0.55 ± 0.33. The mean maximum burn severity for Group 3 (n = 53) was 1.87 ± 0.68, and the mean average burn severity was 1.27 ± 0.58.

### Demographics and timeline

Sex was recorded for all animals, with 104 males and 95 females, with Table 1 showing the distribution between the three bandaging groups. Age was recorded for 174/199 (87.4%) of animals, with 34/174 (19.5%) independent juveniles (1–2 years), 67/174 (38.5%) young adults (3–4 years) and 73/174 (42%) adults (>5 years).

**Table 1:** Sex and age of bushfire‐affected koalas, classified into three burn and bandaging requirement groups

Burn and bandage group^a^	Sex	Independent juvenile	Young adult	Adult	Unknown age
Group 1 n = 61	Male	4	13	8	6
	Female	3	9	13	5
Group 2 n = 77	Male	6	11	16	5
	Female	9	11	14	5
Group 3 n = 61	Male	8	13	12	2
	Female	4	10	10	2

^a^ Group 1: No detectable burn injury; Group 2: Bushfire exposure evident but no triage bandaging; Group 3: Bandaged at triage with significant burn injuries.

**Figure 1:**
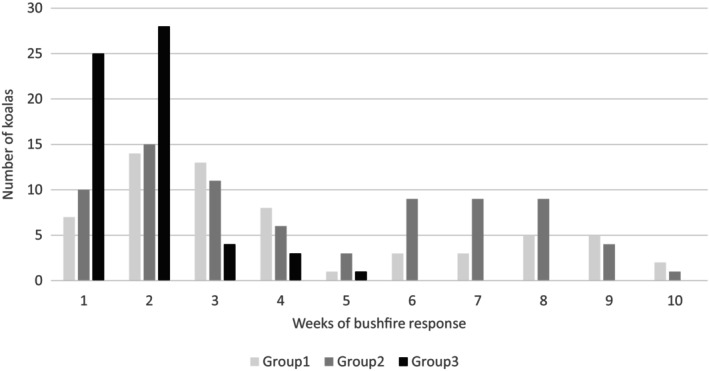
Admissions of koalas from each burn and bandaging group over the duration of the bushfire response. Group 1: No detectable burn injury; Group 2: Bushfire exposure evident but no triage bandaging; Group 3: Bandaged at triage with significant burn injuries.

Based on the date of triage, koalas in Groups 1 and 2 presented over the entire span of the operation, while individuals from Group 3 only presented in the first 5 weeks, with the majority in the first 2 weeks (Figure 1).

The exact hospitalisation period was recorded for 136/199 animals (68.3%) and ranged between 1 and 33 days. When examined by group, the hospitalisation period was not statistically different in length (P = 0.147), with mean total hospitalisation for animals from Group 3 (n = 54) 7.74 ± 6.34 (SD) d, Group 2 (n = 53) 6.45 ±6.74d and Group 1 (n = 29) 5.97 ±5.82 days.

### Overall outcomes

Positive outcomes (release) were seen in 119/199 (59.8%) hospitalised individuals, and negative (death or euthanasia) outcomes in 80 (40.2%) individuals. For positive outcomes, 62 koalas were released to the wild directly from hospital, and 57 koalas entered soft release before release to the wild.

**Figure 2:**
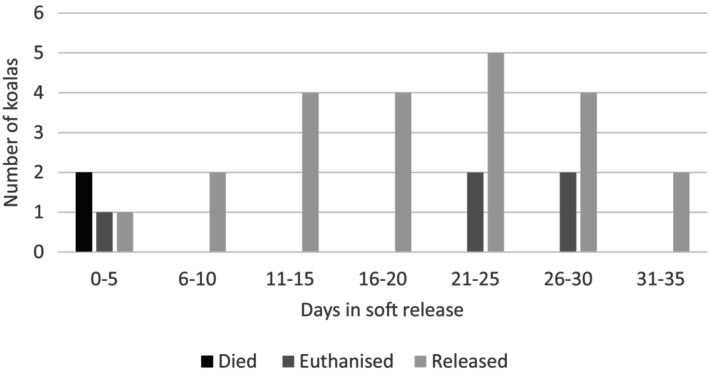
Duration in soft release related to specific outcomes (released, euthanased or died) for bushfire‐affected koalas.

For 29 koalas with adequate soft release data records, there was a mean duration of 18.6 days in soft release. Negative final outcomes occurred in seven of these animals either within the first few days or up to 5 weeks (Figure 2). The fate of koalas released into the wild was beyond the data recorded.

**Figure 3:**
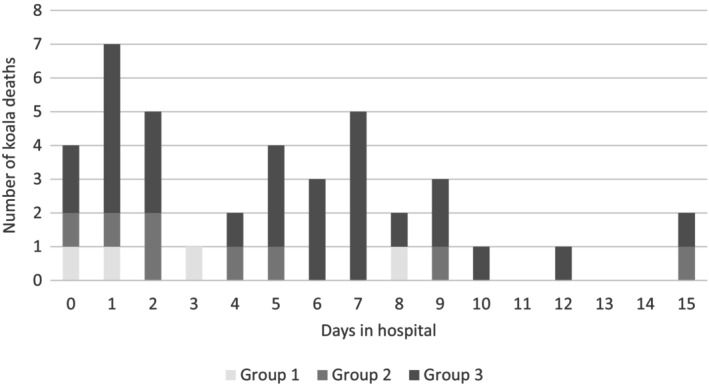
Deaths of bushfire‐affected koalas based on duration in hospital. Group 1: No detectable burn injury; Group 2: Bushfire exposure evident but no triage bandaging; Group 3: Bandaged at triage with significant burn injuries.

For the 80 negative outcomes, spontaneous deaths occurred in 42/80 (52.5%) of animals (40 hospitalised and two in the soft release area) (Table 2), with either no preceding clinical signs or depressed demeanour recorded. Of these animals, 32/42 (76.2%) died within the first 7 days of hospitalisation (n = 40) or rehospitalisation (n = 2) (Figure 3) and 37/42 (88%) within the first 10 days of hospitalisation. Duration to death was unknown for two animals.

**Table 2:** Summary of outcomes (positive or negative) for each group of bushfire‐affected koalas

Burn and bandage group^a^	Positive outcomes^b^	Negative outcomes^b^		
		Died	Euthanased	Total
Group 1 (n = 61)	51 (83.6%)	4 (6.6%)	6 (9.8%)	10 (16.4%)
Group 2 (n = 77)	60 (77.9%)	9 (11.7%)	8 (10.4%)	17 (22.1%)
Group 3 (n = 61)	8 (13.1%)	29 (47.6%)	24 (39.3%)	53 (86.9%)
TOTAL (n = 199)	119 (59.8%)	42 (21.1%)	38 (19%)	80 (40.2%)

^a^ Group 1: No detectable burn injury; Group 2: Bushfire exposure evident but no triage bandaging; Group 3: Bandaged at triage with significant burn injuries.

^b^ Positive outcomes = released; Negative outcomes = died or euthanased.

Of 38 koalas that were euthanased in hospital (including five animals rehospitalised from soft release), 34/38 (89.5%) were euthanased within 3 weeks of presentation. Many of these animals (17/38) were reported as depressed and poorly responsive. Koalas were also euthanased when burns deteriorated (n = 6), body weight declined significantly (n = 2), were found seizuring (n = 4), were anorexic and polydipsic/polyuric (n = 2), presented with significant epistaxis (n = 2) or were significantly aged (n = 1).

### Outcome based on burn and bandaging requirements

Outcomes varied considerably for the three groups (Table 2). Group 1 koalas showed the greatest number of positive outcomes compared to the other two groups, 51/61 (83.6%). Five animals (8.2%) in Group 1 were subsequently diagnosed with burns not requiring bandaging (released n = 3, died in hospital n = 1, euthanased in hospital n = 1). Group 2 koalas also had mostly positive outcomes, with 60/77 (77.9%). Two animals in Group 2 were later recorded as receiving bandages, with both subsequently dying in hospital. The lowest number of positive outcomes, compared to Groups 1 and 2, was seen in Group 3 (8/61, 13.1%) (P < 0.001). Group 3 had the highest number of deaths (29/61, 47.6%) and euthanasias (24/61, 39.3%) compared to both other groups.

The majority of koalas released into the wild directly from hospital were from Groups 1 and 2 (Group 1 n = 24, Group 2 n = 35 and Group 3 n = 3). The three animals from Group 3 released directly from hospital were in the last five koalas to be admitted for treatment in this group. Koalas not directly released from hospital were sent to soft release (SR) areas (Group 1 n = 27, Group 2 n = 25, Group 3 n = 5).

**Figure 4:**
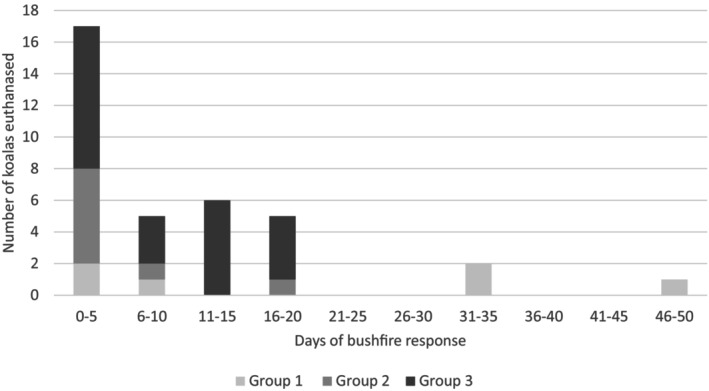
Euthanasia of bushfire‐affected koalas over the duration of the bushfire response. Group 1: No detectable burn injury; Group 2: Bushfire exposure evident but no triage bandaging; Group 3: Bandaged at triage with significant burn injuries.

Based on the hospitalisation timeline, there were a high number of deaths in all groups in the first 15 days. Also, all animals in Groups 2 and 3 that were euthanased had this occur within the first 3 weeks of hospitalisation (Figure 4). Three animals from Group 1 were euthanased after a period of greater than 4 weeks, following their return from the soft release area.

### Outcome based on number of feet burnt

**Figure 5:**
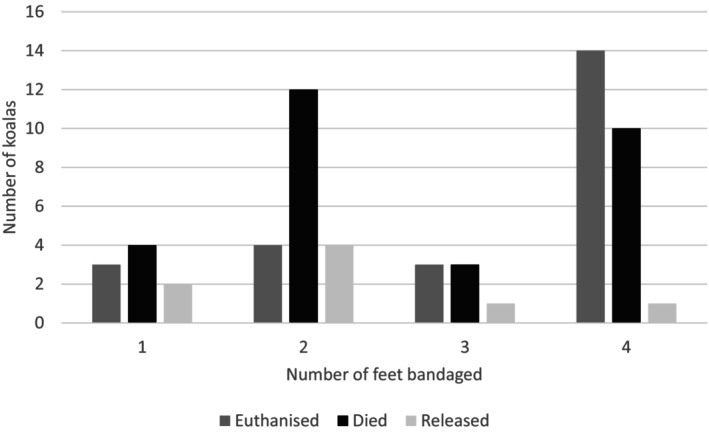
Number of feet bandaged for bushfire‐affected koalas in relation to specific outcomes (released, euthanased or died).

Feet were the most commonly burnt body region, with 129/138 animals presenting with evidence of bushfire exposure (Groups 2 and 3) having one or more feet affected. For those that needed their foot burns bandaged (Group 3), the combination of burns on both forefeet and both hindfeet was most prevalent (25/59; 42%), followed by both hindfeet only (13/59; 22%), and other feet combinations ≤5 koalas. There was a strong negative correlation between the number of feet burnt and positive outcomes (R = ‐0.958, P = 0.042, n = 61), whereby only two of nine (22%) animals with one foot bandaged at triage had positive outcomes, four of 20 (20%) animals with two feet, one animal of seven (14%) with three feet and one of 25 (4%) with four feet bandaged (Figure 5). Seven koalas had injuries to nails/nail beds reported, with one koala later rehospitalised after 4 days in soft release due to the deterioration of a nail injury previously considered healed. Two animals with no apparent nail damage were returned from soft release after 17 and 26 days respectively due to the development of nail and toe injuries suspected to be a sequela to thermal burns.

### Outcome based on number of bandage changes

The number of bandage changes for koalas in Group 3 was also linked to outcomes, whereby no animal (0%) that received more than three sequential bandage changes (maximum six) had a positive outcome. One bandage change resulted in only 25% positive outcomes, two bandage changes resulted in 16% positive outcomes and three bandage changes resulted in 27% positive outcomes. There was also particularly high mortality for koalas following the triage bandage prior to the first bandage change (10 deaths and five euthanasias).

### Outcome based on body condition and hydration scores

Body condition score was recorded at triage for 181 of 199 koalas (Group 1 n = 55/61, Group 2 n = 69/77 and Group 3 n = 57/61). Adequate BCS at triage was associated with more positive outcomes in Group 1 (P = 0.024) and Group 2 (P = 0.048), but not in Group 3 (P = 0.79) (Table 3).

**Table 3:** Body condition score at triage and outcome by group of bushfire‐affected koalas

Body condition score	Koala group^a^	Positive outcomes^b^	Negative outcomes^b^
Adequate (≥3/5)	1 (n = 25)	24 (96%)	1 (4%)
	2 (n = 21)	20 (95.2%)	1 (4.8%)
	3 (n = 14)	2 (14.3%)	12 (85.7%)
	Total (n = 60)	46 (76.7%)	14 (23.3%)
Inadequate (<3/5)	1 (n = 30)	22 (73.3%)	8 (26.7%)
	2 (n = 48)	36 (75%)	12 (25%)
	3 (n = 43)	5 (11.6%)	38 (88.4%)
	Total (n = 121)	63 (52%)	58 (48%)

^a^ Group 1: No detectable burn injury; Group 2: Bushfire exposure evident but no triage bandaging; Group 3: Bandaged at triage with significant burn injuries.

^b^ Positive outcomes = released; Negative outcomes = died or euthanased.

Hydration score was recorded at triage for 163/199 koalas (Group 1 n = 50, Group 2 n = 62 and Group 3 n = 51). Overall, negative outcomes for koalas were correlated with dehydration (R = 0.972, P = 0.028) (Table 4).

**Table 4:** Hydration score at triage and outcome by koala burn and bandaging requirement group for bushfire‐affected koalas

Hydration score	Koala group^a^	Positive outcomes^b^	Negative outcomes^b^
0 (not dehydrated)	1 (n = 24)	20 (83.3%)	4 (16.7%)
	2 (n = 19)	19 (100%)	0
	3 (n = 8)	2 (25%)	6 (75%)
	Total	41 (80.4%)	10 (19.6%)
1 (mild dehydration)	1 (n = 14)	11 (78.6%)	3 (21.4%)
	2 (n = 12)	11 (91.7%)	1 (8.3%)
	3 (n = 8)	3 (37.5%)	5 (62.5%)
	Total	25 (73.5%)	9 (26.5%)
2 (moderate dehydration)	1 (n = 10)	9 (90%)	1 (10%)
	2 (n = 24)	17 (70.8%)	7 (29.2%)
	3 (n = 27)	2 (7.4%)	25 (92.6%)
	Total	61 (45.9%)	34 (54.1%)
3 (severe dehydration)	1 (n = 2)	1 (50%)	1 (50%)
	2 (n = 7)	2 (28.6%)	5 (71.4%)
	3 (n = 8)	0	8 (100%)
	Total	3 (17.6%)	14 (82.4%)

^a^ Group 1: No detectable burn injury; Group 2: Bushfire exposure evident but no triage bandaging; Group 3: Bandaged at triage with significant burn injuries.

^b^ Positive outcomes = released; Negative outcomes = died or euthanased.

### Body weight changes during hospitalisation

**Figure 6:**
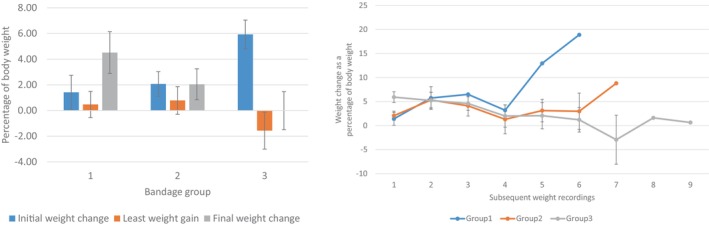
Body weight changes of bushfire‐affected koalas at subsequent weight recordings in hospital, from that recorded at triage. Error bars show SE. Group 1: No detectable burn injury; Group 2: Bushfire exposure evident but no triage bandaging; Group 3: Bandaged at triage with significant burn injuries.

Triage and subsequent hospitalisation weights were recorded for a total of 100/199 koalas (Group 1: n = 21; Group 2: n = 31; Group 3: n = 48) with the maximal changes in body weight ranging from −19.6% to +20.6% (Figure 6). For Group 1, the maximum number of recorded body weight measurements over the period in care for any koala was six (mean ± SE: 1.7 ± 0.3); in Group 2, up to seven (2.6 ± 0.3), whilst in Group 3, up to nine weight measurements were recorded whilst in care (3.9 ± 0.3).

The percentage in change in weight of koalas over subsequent examinations was positively correlated for Group 1 (r = 0.858, P = 0.029), with koalas generally increasing in body weight, and markedly negatively correlated for Group 3 (r = −0.804, P = 0.009), koalas generally decreasing in body weight (Figure 6). Group 2 did not show a statistical correlation between changes in weight (increase or decrease) at subsequent examinations (r = 0.458 P = 0.301).

### Antibiotic usage

Antimicrobials were administered to 5% (3/61) of Group 1 animals, 18% (14/77) of Group 2 animals and 93% (57/61) of Group 3 animals. Of the animals receiving antimicrobials, only 13.5% (10/74: five from Group 2 and five from Group 3) had positive outcomes (8.4% of the 119 animals with positive outcomes).

Amoxicillin (15 mg/kg body weight; Betamox L.A. Injection; Norbrook, Newry, Northern Ireland) was the most frequent antimicrobial administered (n = 68), followed by enrofloxacin (5 mg/ kg body weight, Baytril injection, Bayer Animal Health, Australia) (n = 14), amoxycillin clavulanate (8.75 mg/kg body weight, Noroclav Injection; Norbrook, Newry, Northern Ireland) (n = 10) and procaine and benzathine penicillin (26.5 mg/kg body weight; Duplocillin injection, MSD Animal Health, Australia) (n = 3).

### Fluid therapy

Most animals received subcutaneous balanced electrolytes fluid therapy at triage and subsequent examinations at the clinician's discretion dependent on hydration assessment. This was given at a rate of 5–10 ml/kg, but this was not consistently recorded or quantified in hospital records.

### Analgesics

Analgesics were administered to 11.4% (7/61) of Group 1 animals, 26% (20/77) of Group 2 animals and 95% (58/61) of Group 3 animals. Non‐steroidal anti‐inflammatories meloxicam n = 77 (0.2 mg/kg body weight, Metacam injection; Boehringer Ingelheim, Germany) and flunixin n = 5 (1 mg/kg Ilium flunixil injection; Troy Animal Healthcare, Australia) were used, and opioid analgesics buprenorphine n = 29 (0.01 mg/kg body weight, Temvet Injection; Troy Animal Healthcare, Australia) and tramadol n = 1 (5 mg/kg, Tramal injection; Seqirus, Australia). Often, analgesics were given in combination with each other.

### Laboratory findings

All haematology results (n = 52) were within published reference intervals for koalas,^19^ so they were not of clinical significance. However, both lymphocyte counts and neutrophil counts were statistically higher in Group 3 compared with Group 2 (P < 0.05) and lymphocyte counts were elevated within the reference interval in Group 1 compared with Group 2 (P < 0.05).

The majority of biochemical results (n = 53) were within published reference intervals, with the exception of serum sodium concentration, which was mildly elevated from the reference value range 132–145 mmol/L^20^ in Group 3 (n = 8; 149 ± 6.3 mmol/L), and statistically higher than both Groups 1 (P < 0.001; n = 23; 140.7 ± 3.7 mmol/L) and 2 (P = 0.002; n = 23; 141.4 ± 10.3 mmol/L). Total protein (P < 0.001), globulins (P < 0.001), potassium (P < 0.005) and chloride (P < 0.01) were elevated in Group 3 compared with Groups 1 and 2, but were within published reference intervals for all groups. Serum cortisol concentration was below the threshold of detection in 45/53 koala samples (<27.6 nmol/L) and ranged from 28.4 to 61.5 nmol/L in eight koalas from all three bandage groups.

Full gross necropsy exams were performed on four koalas, with sepsis identified in all four animals and gross evidence of typhlitis in two. In six further koalas, single tissues were taken for histopathology: kidney samples from five koalas, two diagnosed with oxalate nephrosis, and skeletal muscle from one koala with pyogranulomatous necrotising myositis.

### Molecular findings

Conjunctivitis, a known clinical sign of ocular chlamydia, was reported in 17 koalas; however, of the stored swabs available, all koalas tested were negative for *Chlamydia pecorum* (54 ocular swabs and 55 urogenital swabs). KoRV was detected in 25.9% (21/81) animals, and infection status was not found to be associated with group (P = 0.337) or outcome (P = 0.371). Phascolarctid gammaherpesvirus 1 was detected in 2/54 (3.7%) of ocular swabs and 5/55 (9%) of urogenital swabs. Phascolarctid gammaherpesvirus 2 was detected in 3.7% (2/54) ocular swabs from koalas and 1.8% (1/55) urogenital swabs. Only one koala had both types of gammaherpesvirus detected in ocular swabs. Association with koala groups or outcomes was unable to be determined due to the low prevalence.

## Discussion

This study retrospectively analysed clinical record data for 199 koalas that proceeded past the point of triage following the 2019–2020 bushfires on Kangaroo Island. Burn presence and bandaging requirement was utilised as a proxy for acuteness and severity of burns, due to the extended duration of the operation and range of presentations. This grouping resulted in the key findings that negative outcomes increased for koalas that required bandages on admission, and also with the number of subsequent bandage changes and the number of feet that required bandaging. Associations with dehydration, weight loss during hospitalisation and elevated sodium were also significant for bandaged koalas. Furthermore, the timing of koala presentation in relation to the active bushfire window was identified as an important prognostic factor, as high mortality was seen in the first 2 weeks of the operation across all three groups.

Burns severe enough to receive bandaging (allocation to Group 3) were significant factors for negative outcomes, with prognosis being more favourable in Groups 1 and 2. However, in all groups there was mortality, with fewer euthanasia decisions directly related to burn injuries than to signs of generalised systemic disease (depressed demeanour, weight loss, seizures). At least some of this mortality may relate to sepsis or caeco‐colic dysbiosis/typhlocolitis syndrome (CDTS),^21^ oxalate nephrosis^22^ and more generalised conditions such as DIC or multi‐organ failure, presumably as a result of thermal damage. Appreciation of occult bushfire injury such as smoke inhalation was limited by the absence of diagnostic imaging and few necropsies, but this has been shown to be common in bushfire‐affected koalas from other regions.^23^

Severe burns likely to require multiple bandage changes represented an important prognostic factor. Most koalas with positive outcomes were in Groups 1 and 2 and hence had a mean burn score of one or less, indicating that average burn severity could be a significant prognostic indicator and a useful tool at triage. Increasing burn severity ultimately leads to more intensive treatment, handling, potential increased antimicrobial use and longer hospitalisation, which are likely associated with increasing mortality. No animals that received more than three bandage changes following triage had positive outcomes. Mortality seen soon after the initial triage bandaging event was likely due to animals succumbing to the initial insult of bushfire exposure and after the second bandage change following a period of hospitalisation.

Feet were the most frequently burnt areas, and the prognosis for animals with feet burns was worse with an increasing number of feet burnt, or if hindfeet were burnt. The two categories of burn distribution receiving the highest number of bandages were two hindfeet or all four feet burnt. Gaschk et al.^24^ analysed koala gaits over various substrates, including overground, and the fastest strides on the ground were described as bounds and half bounds, with the hindfeet having a greater contact time with the ground than the forefeet. This, combined with the body mass being greater caudally, may predispose the hindfeet to greater pressure and contact time when moving across recently burnt areas, potentially increasing the risk of burns and burns of increased severity. Koalas also sit in a bipedal stance when at rest or in trees, placing the hindfeet in contact with the substrate.

Baek et al.^23^ demonstrated histologically that burn depth was of a higher grade in the hindfeet of koalas than in the forefeet, and nearly all burns were more severe histologically than what was observed clinically. The requirement for hindfeet and forefeet to be bandaged at triage significantly affected prognosis, with only one animal with all feet bandaged at triage resulting in a positive outcome. In addition, it was observed that thermal necrosis of nails and associated tissues may be more difficult to assess clinically at triage, taking more time to become apparent, similar to that found by Baek et al.^23^

Burns are often described based on total body surface area.^11^, ^25^ Vaughn and Beckel^25^ describe burns <20% of total body surface area (TBSA) as ‘local burns’ and those >20–30% as ‘severe burn injuries’. Blanshard and Bodley^11^ refer to unpublished guidelines advising euthanasia with burns >50% of TBSA and/or severe burns affecting the face and/or genitals. With feet being the most common site of burn injury in koalas, injuries could be considered severe even if less than 20% of the TBSA was affected, particularly if nails are lost or damaged, as suggested in recent online guidelines.^26^

Assessment of burns at triage, in some cases, differed from that found during hospitalisation. Five animals in Group 1 were later diagnosed with burn injuries, and two animals from Group 2 later received bandages. This shows that a single assessment at triage is not always sufficient when assessing burns, with the dynamic nature of burns requiring continual reassessment. However, for the majority of koalas presented, the requirement for bandaging at triage served as a proxy for burn severity. Animals that progressed from the initial triage in Group 3 required the most intensive treatment, closest monitoring, and were at most risk of deterioration.

For Group 3, BCS was not statistically associated with prognosis; hence, it is likely other factors had a greater effect on the mortality in this severely affected group. This contrasted with the lesser affected Groups 1 and 2, in which inadequate BCS at triage worsened prognosis, suggesting this factor could be potentially more important when animals do not receive severe burns. Animals presenting in poor body condition may not have been well prior to the fire, affecting their prognosis during treatment and hospitalisation.

Hydration score at triage showed increasing mortality with worsening hydration scores; hence, hydration score at triage represents a significant prognostic indicator. This clinically determined dehydration is supported by the biochemistry findings, with Group 3 having statistically higher total protein and electrolytes than Groups 1 and 2, although they were within the reference intervals. Rehydration therapy is clearly important for animals rescued from bushfire during already hot, dry weather.

Declining body weight while hospitalised was also a significant prognostic factor. Body weight changes while hospitalised showed differences between the three groups, with a significant increase in initial percentage weight change in Group 3 attributed to rehydration therapy. The positively correlated percentage weight gain seen in Group 1 is likely due to minimal intervention in these animals and shorter hospital stays. The negatively correlated percentage weight gains in Group 3 are not unexpected, as these animals were the most intensively treated, and multiple factors such as antimicrobials, stress and debilitation could lead to inappetence and an inability to maintain a positive energy balance. It also highlights the importance of regular weight checks in hospitalised koalas (combined with monitoring hydration status, gut fill, appetite and faecal pellet production), while minimising unnecessary handling.

Gillett and Hanger^21^ suggest welfare and treatment outcomes can be improved with the provision of quiet environments with minimal disturbance. It is difficult to fulfil these ideal requirements in a triage and treatment facility during a mass casualty event. Even with the construction of two soft release areas, which minimised human contact and provided a more natural environment, the less intensive monitoring of koalas allowed for the expression of chronic problems, occult burns and deteriorating nail injuries.

The large number of animals in care (and associated personnel) and the need for frequent repeated procedures to replace bandages were likely important stressors that could influence the development of anorexia, ileus and CDTS.^21^ Whilst blood cortisol at triage was low in almost all koalas, as has been found in studies involving koala capture‐release,^27^ it may have increased over time, reflecting the impact on Group 3 koalas that had longer hospital stays, and subsequent sampling and testing would have been useful.

Antimicrobials were administered to 74/199 koalas, with only 10 having positive outcomes. While these tended to be the more severely burn‐affected animals, and caution is advised with antimicrobial use in koalas, we were unable to determine any contribution antimicrobials may have made to mortality due to the limited number of necropsies. Several systematic reviews of antibiotic use in human burn patients have found that the current evidence (while limited) does not support the use of systemic antimicrobials in the majority of patients.^28^, ^29^ Also of concern is the presence of antimicrobial resistance in the microbiomes of koalas from this operation (identified using cloacal swabs).^30^, ^31^ The risks to koalas of fatal dysbiosis, and the risks of increasing antimicrobial resistance in koala populations must be weighed against the benefit and the appropriateness of their use.

Both non‐steroidal anti‐inflammatory drugs (NSAIDs) and opioids were used for analgesia. The use of opioids in a large‐scale mass casualty situation is problematic with regard to securing and documenting the use of controlled drugs. Because of this, the use of tramadol and buprenorphine tended to be restricted during procedures and were only used by some clinicians. The most used opioid was the mixed agonist/antagonist buprenorphine; generally, this was administered at the time of procedures such as bandage changes. As analgesic drugs were used most extensively in the more severely affected Group 3, it is difficult to determine the advantages of drug combinations or one drug over another.

Meloxicam, the most used NSAID, is accessible and does not require documentation; however, studies have shown it may have limited efficacy in koalas due to hepatic metabolism and a short half‐life.^32^ Analgesics were generally used at the time of procedures and even this short duration, particularly when administered by the subcutaneous route, would likely have had some anti‐inflammatory and analgesic effect. The use of meloxicam in animals with some degree of dehydration (at the time of rehydration therapy) represented one of many compromises in a mass casualty situation. In smaller‐scale operations or at existing koala hospital facilities, more frequent use of opioid analgesics would be recommended, but this would necessitate appropriate recording, and close monitoring and treatment of adverse effects. The choice of other non‐steroidal analgesics should ideally be based on a better understanding of their pharmacokinetics in this species.

Overall, in this spontaneous bushfire response, there was an evolution of hospital infrastructure, triage facilities, resources, treatment protocols and data recording, making retrospective data analysis challenging. Over 11 weeks, the ability of the centre to respond improved, for example, koalas could rehabilitate in an outdoor naturalistic soft release area rather than small, indoor temporary enclosures. Also, the number of koalas presented and the severity of injuries both decreased. The use of paper records created problems with records being lost, damaged or soiled as animals were moved around the facility. An attempt was made to group information recorded by different clinicians as accurately as possible in a way that meaningful conclusions can be derived.

Whilst the utilisation of bandaging at triage as a proxy for burn injury acuteness and severity should be recognised as somewhat problematic as it relied on the decisions of 22 different clinicians, and certain areas do not easily allow bandages to be applied, the approach did allow the distinction between acutely burnt animals and those presenting later with healed burns. This then allowed identification of prognostic factors associated with outcomes in affected koalas.

Bushfire events are frequently occurring but unpredictable; hence, it is likely that being fully prepared for a mass casualty event may be impossible. However, in areas with substantial bushland and large numbers of wildlife, it may be useful to undertake planning and to enter discussions with stakeholders prior to any event. Defining goals and developing record‐keeping systems that allow consistent recording of information beforehand would enable more effective and realistic triage of wildlife casualties. Combined with further studies into prognostic indicators, this could improve welfare outcomes and maximise the use of resources. In this operation, like many similar operations, post‐release monitoring of recovered animals becomes difficult, although this is arguably a vital measure of the effectiveness of an operation. The impact that treated and recovered animals could have both on the unaffected population and the health of the population and wider environment should be factored into planning.

By encouraging a consistent way of recording the triage and treatment of bushfire‐affected koalas nationally, and rigorous analysis of data, it may be possible to discover trends to refine both triage and treatment of bushfire‐affected koalas. This could be aided by concentrating efforts, where possible, on designated centres and utilising trained and experienced staff. SAVEM represents a significant change in the rapid mobilisation and organisation of large numbers of skilled volunteers. In this operation, this was integrated with staff from other organisations (ZOOSSA, RSPCA, KI Wildlife Park and KI Veterinary Clinic, Australian Defence Force and others) with specialist skills, and importantly, was ultimately managed and overseen by local stakeholders (KI Veterinary Clinic and KI Wildlife Park).

This study found that negative outcomes of bushfire‐affected koalas were associated with the timing of presentation, admission hydration scores, decreasing hospital body weights, an increasing number of bandage changes during hospitalisation and burns on all four feet. The prognostic factors identified in this study should assist in developing evidence‐based protocols to enable realistic, accurate, consistent and efficient decision‐making on the front line and enable targeting of resources, especially in mass casualty situations for koalas.

## Conflict of interest and sources of funding

The authors declare no conflicts of interest. This project was in part funded by the Morris Animal Foundation Australia Wildlife Fund grant ID: D21ZO‐506 awarded to NS; and in part by the Department of Agriculture, Water, and the Environment, Australian Government, and Prague Zoo, awarded to Zoos South Australia.

## Data Availability

The data that support the findings of this study are available from the corresponding author upon reasonable request.
